# Adherence-related factors associated with weight reduction: clinical outcomes of telerehabilitation in patients with metabolic syndrome

**DOI:** 10.3389/fcvm.2026.1804026

**Published:** 2026-10-26

**Authors:** Judit Erzsébet Ábrahám, Anna Vágvölgyi, Éva Máthéné Köteles, Andrea Korom, Mária Barnai, Mónika Szűcs, Brigitta Szálka, András Király, István Vassányi, Attila Nemes, Tamás Várkonyi, Csaba Lengyel, István Kósa

**Affiliations:** 1 Department of Preventive Medicine, Albert Szent-Györgyi Medical School, University of Szeged, Szeged, Hungary; 2 Department of Medicine, Albert Szent-Györgyi Clinical Center, University of Szeged, Szeged, Hungary; 3 Department of Physiotherapy, Faculty of Health Sciences and Social Studies, University of Szeged, Szeged, Hungary; 4 Department of Medical Physics and Informatics, Albert Szent-Györgyi Medical School, University of Szeged, Szeged, Hungary; 5 Medical Informatics Research and Development Centre, University of Pannonia, Veszprém, Hungary

**Keywords:** metabolic syndrome, nutrition, telemedicine, telerehabilitation, training

## Abstract

**Background:**

Metabolic syndrome (MetS) is a major contributor to cardiovascular morbidity, yet our healthcare system is underdeveloped in terms of effective lifestyle interventions. Telemedicine-based programs may offer a scalable option, but evidence on their effectiveness in MetS populations is limited.

**Objective:**

To evaluate a combined telemedicine-supported lifestyle program compared to non-randomized controls in adults with MetS and identify adherence-related factors associated with weight loss.

**Methods:**

In this prospective, non-randomized controlled intervention study (2021–2023, Hungary), 133 adults with MetS were enrolled and 111 completed a 90-day lifestyle program. All participants received an initial dietetic consultation; the intervention group additionally used smartphone-based dietary logging, remote exercise monitoring, and biweekly teleconsultations with dietitians and physiotherapists. Primary outcome was waist circumference at the standardized day-90 follow-up; secondary outcomes included body weight, body composition, exercise capacity, metabolic parameters, and adherence indicators. Outcomes were analyzed by baseline-, age-, and sex-adjusted ANCOVA with HC3-robust standard errors; the primary model included a group-by-baseline waist circumference interaction, and exploratory adherence analyses assessed linear trends across ordered weight-loss subgroups.

**Results:**

A significant group-by-baseline waist circumference interaction was observed (*p* = 0.029), with a greater adjusted intervention–control difference at higher baseline waist circumference. The intervention group also showed descriptive reductions in body weight (–5.23 ± 5.16 kg), BMI, skinfold thicknesses, body fat mass, and visceral fat. HbA1c declined by 0.41 ± 0.60 percentage points (*p* < 0.001), and HDL-cholesterol increased significantly, while other laboratory parameters changed modestly. After Benjamini–Hochberg adjustment within the respective outcome families, the adjusted between-group differences in abdominal circumference, HbA1c, and skeletal muscle mass remained statistically significant. Exploratory adherence analyses showed significant trends toward more frequent body-weight logging and lower relative energy intake with greater weight loss, but not for the other adherence measures.

**Conclusions:**

A telemedical lifestyle program was associated with favorable short-term changes in abdominal obesity, body composition, and metabolic control in adults with MetS, although skeletal muscle mass loss was also observed. Regular self-weighing and maintaining a lower relative energy intake were associated with greater weight loss. Telemedicine-supported lifestyle management may represent a promising approach for MetS care.

## Introduction

According to the Obesity Atlas (2024), the number of adults living with obesity was 0.81 billion worldwide in 2020, and this figure is projected to rise to 1.53 billion by 2035 (1) Overweight is currently responsible for more than 120 million adult person-years lost due to four leading non-communicable diseases — diabetes, stroke, coronary heart disease, and cancer. There is a clear correlation between the prevalence of obesity and that of diabetes in different populations (2). The clinical relevance of obesity is increasing in cardiology, as it increases the risk of coronary artery disease (CAD) by 49% and the risk of heart failure by 96% (3). Metabolic syndrome (MetS) is a special condition in which the co-existence of three or more risk factors typically related to overweight (abdominal obesity, abnormal glucose metabolism, abnormal triglyceride or HDL-cholesterol metabolism, and hypertension) predicts an elevated risk for the development of a cardiovascular event (4, 5). According to a recent systematic review, patients with MetS have a 4.03-fold higher risk of developing CAD (6). Cross-sectional studies of patients from invasive as well as non-invasive cardiac departments have established the prevalence of MetS to be as high as 47.3–49.5% (6, 7). The presence of MetS in patients with known CAD is coupled with about a 50%–80% increased risk of cardiovascular mortality as well as major cardiovascular events (7, 8), so special attention to the control of this condition is clearly important even in patients with established CAD.

Given the substantial social and financial burdens associated with MetS, there is a global effort to develop new management strategies, including lifestyle modification, pharmacological therapy, and bariatric procedures (9–11). Lifestyle clearly plays a pivotal role in the development and progression of MetS; however, lifestyle modification represents a labor-intensive intervention that typically requires at least 7–10 face-to-face sessions between the patient and healthcare professionals over an intervention period of 3–12 months (12).

Physical exercise training alone can have beneficial effects on the parameters of patients with MetS if practiced for more than three months (13). Our working group and others have demonstrated that wearable gadgets such as step counters and ECG- or optical-based heart rate monitors can effectively support coaching for home-based physical activity with moderate human effort (14–16).

Diet represents a crucial modifiable component of lifestyle for patients with MetS. Both scientific evidence and clinical experience indicate that interventions consisting of only a few consultations are generally ineffective in promoting sustained weight loss. Instead, successful and durable lifestyle changes require comprehensive programs that include at least one session per month (17, 18) or ideally more than 14 sessions within a six-month period to achieve optimal results (19). Such intensive programs demand significant time and financial investment from both healthcare providers and patients.

Self-management interventions, such as dietary self-monitoring can alone support the process of lifestyle change in overweight patients (20). Dietary self-monitoring using digital services, such as web or mobile applications, is more effective than paper-based food diaries (21). This enhanced effectiveness can be attributed to the immediate feedback provided by digital platforms, which enables users to receive precise information regarding the balance of their calorie consumption and estimated energy requirements. Theoretically, locally generated digital food diaries are suitable for telemedical consultations, but the integration of such data in the workflow of medical services is limited, because most digital food diary services have data protected by contract law. On the other hand, the added value of teleconsultation based on locally generated digital diary data remains uncertain, primarily because of the heterogenity of the feedback provided (22, 23). Typically, personalized feedback may confer approximately a 2 kg benefit over interventions that did not provide personalized feedback (24).

The University of Szeged has a long-standing research collaboration with the University of Pannonia. Within the framework of this collaboration, we had the opportunity to expand the training monitoring study previously launched at the University of Szeged (16, 25) with a university-owned mobile nutrition logging service.

## Aims

The current study aimed to evaluate the effects of a combined exercise and dietary intervention on the anthropometric parameters, functional capacity, and laboratory parameters of MetS patients. Additionally, we sought to identify the factors associated with the favorable outcomes of this integrated telemedical program.

## Materials and methods

### Study design and patient recruitment

This research was a prospective, non-randomized controlled intervention study conducted on patients with MetS between July 7, 2021, and June 24, 2023. Participants were drawn from the city of Szeged, Hungary, and nearby villages within a 40 km radius. Recruitment took place in general practitioners’ and occupational physicians’ offices, as well as in cardiology inpatient and outpatient facilities. The participants joined the study through physician referrals. All individuals were informed about the study procedures and provided written consent prior to enrolment. The study was approved by the Ethics Committee of the National Institute of Pharmacy and Nutrition of Hungary in 2020 (No. OGYÉI/46241/2020) based on the prior favorable opinion of the Hungarian Medical Research Council (ETT TUKEB; Ethical Trial Number: 50780-2/2017/EKU). The study was designed and conducted in accordance with the principles of the Declaration of Helsinki (1975, as revised in 2000). The clinical trial was registered at ClinicalTrials.gov under the identifier NCT05117580.

The study deviated from the registered protocol (NCT05117580) with respect to the study design and the definition of the primary outcome. First, the original study design was modified due to the advent of the COVID-19 pandemic, which rendered randomization of patients into the control arm infeasible. Consequently, participants were enrolled non-randomly and sequentially over time, with the active group recruited first and the control group recruited after the pandemic. The pandemic also affected the timing of the follow-up assessments, which were performed between 83 and 178 days after enrolment. To account for variation in follow-up duration, follow-up values were standardized to day 90 using the following linear calculation: 90-day value = baseline value + (observed follow-up value−baseline value) × 90/follow-up duration in days. Follow-up occurred after day 90 in 94 participants, for whom interpolation was applied, and before day 90 in 12 participants, for whom extrapolation was applied. Five participants were assessed exactly on day 90 and therefore required no temporal adjustment. The standardized 90-day values were used in the primary analyses.

Second, the definition of the primary outcome differed from that specified in the registered protocol (NCT05117580). The protocol was developed based on our previous publication, in which the term waist circumference was used for measurements obtained at both the midriff and umbilical levels. In the present study, waist circumference measured at the midriff level was retained as the primary anthropometric outcome because it was the variable used for the original *a priori* sample-size calculation and because it corresponded to the measure used to define abdominal obesity according to the metabolic syndrome criteria applied in this study. Abdominal circumference, measured separately at the level of the umbilicus, was retained and analyzed as a secondary outcome.

Inclusion criteria:

- Age between 18 years and 75 years
- Presence of at least three of the following five risk factors (5)
- Waist circumference above 102 cm in men and 88 cm in women
- Treated diabetes or fasting blood glucose above 5.6 mmol/L
- Treated hypertension or spontaneous blood pressure ≥ 130/85 mmHg
- Fibrate treatment or triglyceride levels above 1.7 mmol/L
- HDL cholesterol levels below 1.03 mmol/L in men and 1.3 mmol/L in women
- Provision of written informed consent
- Ability to communicate effectively with the investigator and to understand and comply with the study protocol

Exclusion criteria:

- Low physical activity level (less than 30 min per day)
- Insufficient IT proficiency for basic mobile phone use (e.g., placing or receiving voice calls)
- Planned invasive cardiology intervention (e.g., catheter-based vasodilatation, coronary artery bypass grafting, valvuloplasty, or valve replacement)
- Uncontrolled high blood pressure (BP >160/100 mmHg)
- Type 2 diabetes treated with more than one daily insulin dose
- Type 2 diabetes with heart failure or kidney failure [estimated glomerular filtration rate (eGFR) < 60 mL/min]
- Tumor disease
- Severe cognitive dysfunction
- Lack of cooperation, known conditions preventing physical training, or conditions severely impairing insight or functional capacity

### Patient evaluation and preparation

At the commencement of the study, patients were admitted to the day-care unit of the Cardiometabolic Rehabilitation Department of Medicine at the University of Szeged. A physician reviewed and documented the detailed medical history, available laboratory data, and current drug treatments during the initial medical evaluation. Using this information, together with the assessment of the patients’ physical status, the investigator verified the inclusion and exclusion criteria. The patients received structured instruction in the use of the telemonitoring system, followed by supervised familiarization with the device.

### Anthropometric parameters

The methods used to record anthropometric parameters have been published previously by our working group (16). Height was recorded in centimeters by physiotherapists with a wall-mounted metric stadiometer; waist circumference (WC) was measured in centimeters at the narrowest part of the waist; abdominal circumference (AC) was measured in centimeters at navel level; and hip circumference (HC) was taken at the level of the greater trochanter. All circumference measurements were performed with the participant standing upright, at the end of a normal expiration. Blood pressure measurement was performed after a 3-minute rest in a seated position by an automated blood pressure monitor (Omron M3 Comfort, IntelliSense technology; Omron Healthcare Co., Ltd., Kyoto, Japan).

### Skinfold measurements

Skinfold measurement is a simple, non-invasive method used to evaluate nutritional status and estimate body fat percentage. Subcutaneous tissue thickness was measured at standardized anatomical sites using a mechanical skinfold caliper. The biceps skinfold (vertical fold) was assessed at the midpoint of the anterior aspect of the upper arm, directly over the belly of the biceps muscle. The triceps skinfold (vertical fold) was measured on the posterior surface of the upper arm, along the midline, over the triceps muscle. The site was located halfway between the olecranon and acromion processes, ensuring that the long axis of the lifted skinfold was aligned with the longitudinal axis of the upper arm. The subscapular skinfold (diagonal fold) was obtained approximately two fingerbreadths (1–2 cm) below the inferior angle of the scapula. The skinfold was raised at an angle of roughly 45° relative to the body's midline. The suprailiac skinfold (diagonal fold) was measured at the level of the spina iliaca anterior superior, directly above the anterior, downward-curving portion of the iliac crest. All measurements were performed on the right side of the participant's body while they were standing in an upright position (26).

### Body composition

Body composition was analyzed using a bioelectrical impedance analyzer (InBody 770, Model: BPM040S12FXX, Bridge Power Corp.), measuring the following parameters: body fat mass (BFM), the total mass of fat in the body (kg); percent body fat (PBF), the ratio of body fat to total body weight (%); fat-free mass (FFM), the mass of all non-fat tissues (kg), including muscle, bone, organs, and water; visceral fat area (VFA), a parameter characterizing abdominal fat content (cm^2^); skeletal muscle mass (SMM), the mass of skeletal muscle responsible for voluntary movement (kg); and fat-free mass index (FFMI), a relative measure of fat-free mass adjusted for height (kg/m^2^).

### Cardiovascular assessment and exercise testing

Resting echocardiography was performed using the Canon Xario 100G (Canon Medical Systems, Otawara, Tochigi, Japan) to assess potential contraindications for exercise training.

Maximal exercise capacity and exercise time were assessed using a GE T2100-ST2 treadmill electrocardiogram (ECG) with Cardiosoft software (GE Medical Systems, Milwaukee, WI, USA) under the supervision of a cardiologist, using the modified Bruce protocol of incremental stress. Exercise was continued until the maximum heart rate was reached or the subject became exhausted. The age-predicted maximum heart rate was calculated using the 220−age formula. Maximal capacity in metabolic equivalent of task (METs), time to maximal exercise capacity (s), maximal heart rate (bpm), and systolic and diastolic blood pressure (mmHg) were documented.

### Laboratory parameters

Additionally, detailed laboratory tests were carried out, including fasting blood glucose, HbA1c, total cholesterol, LDL-cholesterol, and HDL-cholesterol.

Patient assessment was repeated using identical measures after completion of the home-monitored intervention at the closing visit.

## Intervention

### Dietary intervention

Patients in the intervention group as well as patients of the control group received an initial individual dietetic consultation to plan their dietary intervention, following accepted principles of medical nutrition therapy (27, 28). The consultation included gathering a detailed dietary history, examining patients’ lifestyle habits, dietary preferences, and consumption patterns. Based on this information, personalized nutritional plans with both short-term and long-term goals were developed collaboratively. Short-term goals included tasks such as increasing vegetable intake, while long-term objectives involved targets such as weight loss. Generally, the plans were flexible and did not prescribe strict dietary restrictions.

The control group received standard institutional care, which commenced with an initial individual dietetic consultation to develop a personalized, flexible nutritional plan based on medical nutrition therapy principles. Physical activity instructions—including type, frequency, duration, and intensity—were identical to those provided to the intervention group. However, follow-up for control participants was limited to a maximum of two in-person consultations as clinically indicated, and they did not have access to smartphone-based logging, remote monitoring, or biweekly teleconsultations.

Patients in the intervention group received a smartphone equipped with a dietary logger and lifestyle app from a previous project (29). The app provided instant visual feedback on the nutritional content of meals relative to personalized recommendations. All data were stored in the InClouded cloud-based database of the University of Szeged, accessible to supervising dietitians for monitoring. Biweekly personal teleconsultations allowed patients to discuss progress, dietary changes, and obtain advice on various situations. During these teleconsultations participants were encouraged to practice self-care, such as preparing healthy snacks for work. The typical time investment was 10–15 min per teleconsultation, not exceeding a total of 90 min during the three months coaching period.

### Home-based physical training

Physiotherapists assessed the patients’ physical activity habits, discussed the facilities available in the home environment for performing regular training, and, using the information from the completed stress ECG, conducted a supervised training session at the institution. Then they instructed the patient to perform 3–5 training sessions per week at home, aiming for at least 150 min weekly over 12 weeks, with freedom in choosing activities (e.g., jogging, running, biking). Recommendations were given on beneficial types and intensities of exercise for MetS patients. The target heart rate zone was individually calculated based on exercise capacity, aiming for 60%–80% of the maximal predicted heart rate. Patients were advised to maintain this target during their activities.

Patients of the active group received a Polar M430 (Polar, Finland) GPS running watch connected to an Android smartphone with a fitness app (Polar Beat, Polar Finland) for home-based training monitoring. Participants received an information sheet and a 15–30-minutes educational session on using their training monitor devices and data transfer procedures. Additional details on devices and measurement procedures were published in our earlier publication (16). The instructions regarding the type, frequency, duration, and intensity of training were identical in the active and control groups.

The Polar device is commercially available at reasonable prices and has the capability to store detailed training data in Polar's cloud-based platform using a unique identifier. Training data were synchronized from this commercial cloud database to the InClouded cloud-based database of the University of Szeged. This was the platform where the physiotherapist was able to identify the patient and perform monitoring. Physiotherapists conducted biweekly teleconsultations with the patients to discuss progress and guide further training sessions. Individual consultations lasted approximately 10–15 min per teleconsultation, not exceeding a total of 90 min during the three-month coaching period. The device logs all daily activities as well as heart rate-monitored training activities. In the statistical evaluation, only data with “training” labels were included.

### Body-weight telemonitoring

Patients of the active group also received a wireless body weight scale and a GSM mobile hub with the capability to transfer home-measured data without patient interaction to the InClouded cloud-based telemedicine system of the University of Szeged. Home-measured body weight data were used both during dietitian and physiotherapist coaching.

### Data cleaning and calculation of derived parameters

Logged food caloric intake and training-related energy expenditure were expressed in relation to basal metabolic rate (BMR, kcal). The equation used for the BMR calculation is the Harris-Benedict equation revised by Roza and Shizgal in 1984 (30).

The calorie and nutrient data logged by the patients were manually supervised by a dietitian. Outlier data were excluded as published earlier (31).

The cleaned dataset was used to identify key parameters in terms of characterizing the adherence of the patients to telemonitoring. These were:

- Ratio of days with food record (food_count_per_duration): the number of days in which any dietary logging was performed by the patient, divided by the number of monitored days.
- Ratio of days with a training record (polar_count_per_duration): calculated as the number of days (polar_count) in which the Polar watch logged data labeled as “training”, divided by the number of monitored days.
- Ratio of days with a body weight record (weight_count_per_duration): calculated as the number of days (weight_count) in which body weight data were logged by the body weight scale, divided by the number of monitored days. This variable was used as a measure of adherence to home body-weight recording.
- Average daily logged calorie intake relative to BMR (food_kcal_mean_per_bmr): calculated as the average daily logged calorie intake divided by the estimated BMR.
- Average daily training energy expenditure relative to BMR (polar_kcal_mean_per_bmr): calculated as the average daily estimated energy expenditure of activities labeled as “training”, divided by the estimated BMR.

### Subgroup analysis

To identify factors correlating with the effectiveness of the intervention, a subgroup analysis was also performed for four subgroups of the active arm based on the relative change of the 90-day adjusted office body weight values. The first subgroup, TQ1, comprised participants who experienced a substantial weight reduction of 7% or more (≥7%). The second subgroup, TQ2, included individuals who achieved moderate weight loss, with a reduction ranging from 5% to 7% (≥5% but <7%). The third subgroup, TQ3, consisted of those who experienced modest weight loss, with a decrease between 2% and 5% (≥2% but <5%). Finally, the TQ4 subgroup was composed of individuals whose weight reduction was less than 2% or who showed no weight loss at all.

### Primary and secondary outcome measures

We established waist circumference as the primary outcome measure, because this is an essential anthropometric measurement for assessing abdominal obesity, which, by definition, is a risk factor for MetS. All other parameters, including various anthropometric, body composition, and metabolic metrics such as HC, body weight, body mass index, maximal exercise capacity (MET), and laboratory results (fasting plasma glucose, glycated hemoglobin, triglycerides, HDL, and total cholesterol levels), were considered secondary outcome measures.

### Statistical methods

*A priori* sample-size calculation performed during study planning was based on the original hypothesis that waist circumference would decrease more in the active than in the control group. Based on previous data (16), a mean change of 2.30 cm (SD 4.38 cm) was assumed in the telemedicine group and no change in the control group. Assuming an ANOVA model followed by Dunnett's test, 80% power, a 95% confidence level, and 10% dropout, 130 participants (65 per group) were planned to obtain 116 evaluable participants. The study objective was revised after completion of data collection but before the statistical analysis began; the originally determined target sample size was retained and no new sample-size calculation was performed for the revised study objective. Continuous variables were expressed as mean ± SD and categorical variables as frequency and relative frequency. Distributional characteristics were assessed using the Shapiro–Wilk test together with inspection of histograms and normal Q–Q plots. Within-group changes from baseline to the day-90-standardized values were assessed using a studentized nonparametric bootstrap test applied to paired differences, with 50,000 resamples and two-sided *p*-values. Clinical outcomes standardized to day 90 were compared between the active and control groups using analysis of covariance (ANCOVA) within a linear regression framework, adjusted for the baseline value of the respective outcome, age, and sex. The waist-circumference model additionally included a group-by-baseline waist circumference interaction; conditional adjusted active-control differences were estimated at the 25th percentile, median, and 75th percentile of baseline waist circumference within the common range of the two groups. The 22 secondary outcomes were analyzed using separate ANCOVA models. Adjusted active–control differences were reported with 95% confidence intervals, with two-sided *p*-values based on HC3 heteroskedasticity-robust standard errors. To account for multiple testing, the secondary outcomes were grouped into clinically related outcome families (anthropometric parameters, body composition parameters, functional capacity, and laboratory parameters), and *p*-values for the adjusted between-group comparisons were corrected within each family using the Benjamini–Hochberg procedure to control the false discovery rate at 5%. As a sensitivity analysis for the waist-circumference outcome, the principal model was repeated using the actual observed follow-up value without temporal standardization. The model included baseline waist circumference, group, age, sex, actual follow-up duration, and the group-by-baseline waist circumference interaction, with HC3 heteroskedasticity-robust standard errors used for inference.

Linear trends across the ordered TQ1–TQ4 subgroups were tested by assigning ordinal scores to the subgroups (TQ1 = 0, TQ2 = 1, TQ3 = 2, TQ4 = 3) and fitting a linear regression model. HC3 heteroskedasticity-robust standard errors were used for inference on the trend. Pairwise subgroup comparisons were adjusted using the Holm method; these analyses were considered exploratory.

Distributions and model assumptions were assessed using the Shapiro–Wilk test and graphical residual diagnostics. Complete-case analyses were performed without imputation. All tests were two-sided; *p* < 0.05 was considered statistically significant (16). Statistical analyses were performed using R, version 4.4.0 (R Foundation for Statistical Computing, Vienna, Austria).

## Results

### Subject population

The participant flow, including screening, enrollment, allocation, and the reasons for exclusion or dropout from the final analysis, is illustrated in the CONSORT-style flow diagram (Figure 1). The study included 133 patients with MetS, 65 men and 68 women, with a mean age of 57.9 ± 10.41 years. Twelve participants (18.2%) from the control group did not return for the follow-up examination, while 9 participants in the active arm discontinued participation during the intervention, and one participant had to stop the intervention early because of orthopedic surgery (10 participants, 14.9% dropped out from the active arm). No adverse events were reported to the study staff during the home exercise program or at the final assessments. Furthermore, medication regimens remained stable throughout the study, with no documented changes in drugs affecting body weight, carbohydrate metabolism, or lipid profiles in either study arm. A total of 111 participants completed follow-up and constituted the main analysis cohort (57 in the active group and 54 in the control group). Follow-up duration was 113.7 ± 29.4 days in the active group, with a median of 98.0 days (IQR 91.0–137.0; range 83–178), and 98.9 ± 14.1 days in the control group, with a median of 97.5 days (IQR 91.0–101.0; range 83–155). Their baseline characteristics are shown in Table 1. Because the non-randomized groups differed in several baseline characteristics, the principal between-group analyses compared the active and control groups using models adjusted for the baseline value of the respective outcome, age, and sex.

**Figure 1:**
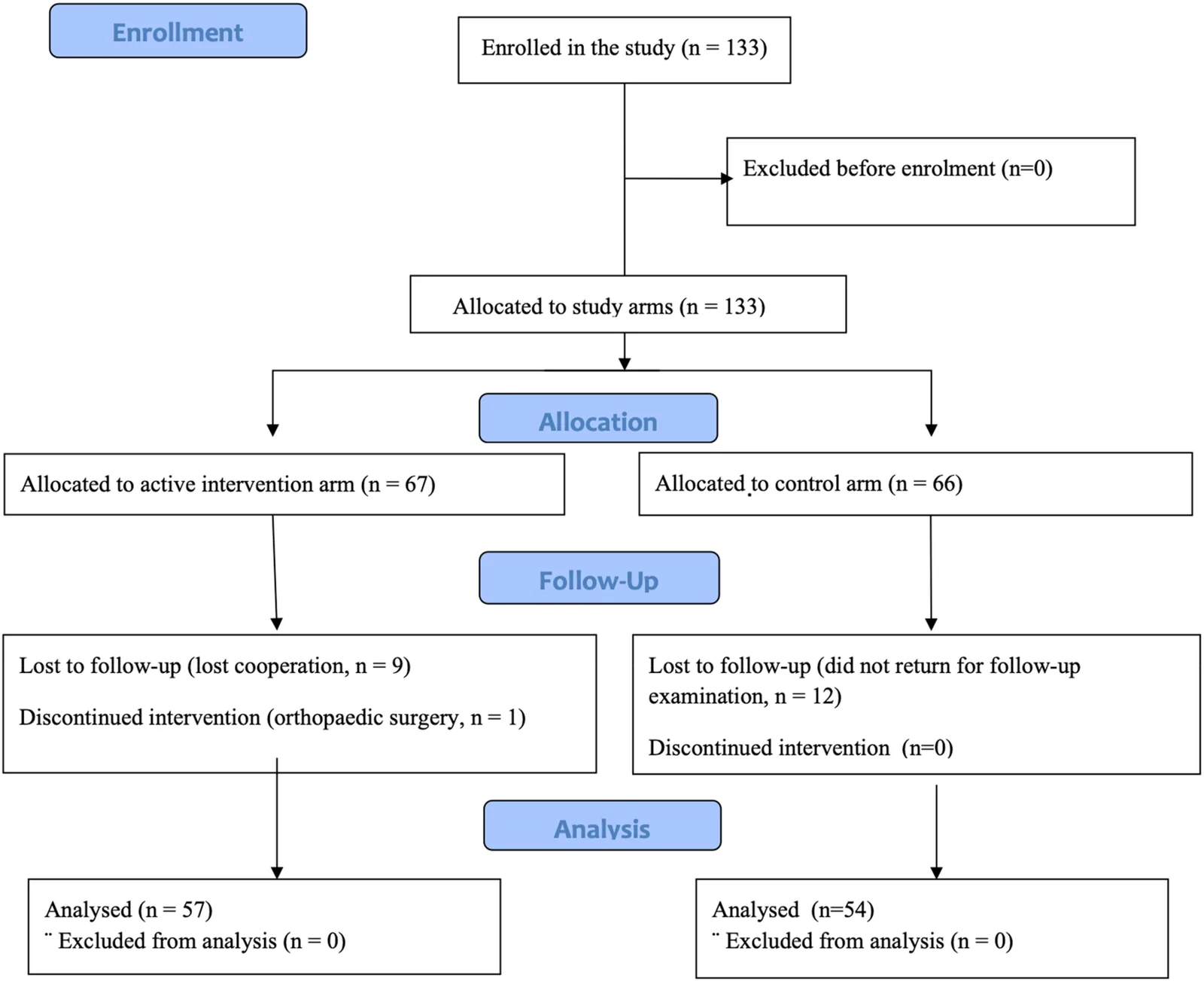
Flow of patients through the telemedical lifestyle intervention trial.

**Table 1:** Baseline patient characteristics.

Characteristic	Active group (*n* = 57)	Control group (*n* = 54)	*P*-values
Gender			0.999
Male	28 (49.1%)	26 (48.1%)	
Female	29 (50.9%)	28 (51.9%)	
Age *(years)*	55.6 ± 10.33	62.1 ± 7.99	**<0**.**001**
Weight *(kg)*	113.4 ± 32.73	98.0 ± 21.99	**<0**.**001**
Waist circumference *(cm)*	116.70 ± 19.98	107.77 ± 12.10	**0**.**016**
Basal metabolic rate *(kcal)*	1986.5 ± 551.32	1746.6 ± 374.56	**0**.**008**

Bold *p*-values indicate statistically significant differences between the active and control groups at baseline (*p* < 0.05).

### Logged parameters during telemedical coaching

In the intervention arm, patient logged their food intake on 74.1 ± 22.60% of the monitored days. The average daily logged calorie intake was 1,463.9 ± 244.3 kcal, which was 77.1 ± 20.59% of the estimated BMR of the patients. Patients logged their training activity on 47.3 ± 24.09% of the monitored days. Because the program prescribed 3–5 training sessions per week rather than daily exercise, adherence relative to the prescribed weekly training frequency was 82.77%. The average estimated calorie expenditure of logged training was 548.0 ± 415.18 kcal, which was 25.2 ± 20.83% of the estimated BMR of the patients. Patients measured their body weight on 71.8% ± 24.91% of days.

### Outcome measures

The primary outcome measure, WC, decreased significantly in the active group (mean change −4.98 ± 6.91 cm, *p* < 0.001), but not in the control group (−1.17 ± 3.88 cm). The adjusted analysis showed a significant group-by-baseline WC interaction (HC3 *p* = 0.029), indicating that the between-group difference depended on baseline WC. At baseline WC values of 100.1, 108.0, and 118.1 cm, corresponding to the 25th percentile, median, and 75th percentile, the adjusted active–control differences were 0.08 cm (95% CI −1.99 to 2.14), −1.67 cm (95% CI −3.33 to 0.00), and −3.90 cm (95% CI −6.73 to −1.07), respectively. Thus, the between-group difference increasingly favored the active group at higher baseline WC values (Table 2). The sensitivity analysis using the actual observed follow-up values showed a similar baseline-dependent pattern (group-by-baseline waist circumference interaction, HC3 *p* = 0.032). At a baseline waist circumference of 118.1 cm, the adjusted active–control difference was −4.75 cm (95% CI −8.02 to −1.48; HC3 *p* = 0.005), compared with −3.90 cm (95% CI −6.73 to −1.07; HC3 *p* = 0.007) in the main day-90-standardized analysis.

**Table 2:** Outcome measures I. Anthropometric and body composition parameters for the whole study population (active and control groups).

Parameter	Active group					Control group					Adjusted between-group analysis	
	n	Pre	Post	*Δ*	Pre-Post *p*-value	n	Pre	Post	*Δ*	Pre-Post *p*-value	AACD (95%CI)	BH-adjusted *p*-value
Anthropometric parameters												
Waist circumference (WC) *(cm)*	57	116.70 ± 19.98	111.72 ± 16.03	−4.98 ± 6.91	**<0**.**001**	54	107.77 ± 12.10	106.60 ± 12.73	−1.17 ± 3.88	**0**.**051**		**0**.**029**^a^
Baseline WC = 100.1 cm											0.08 (−1.99 to 2.14)^a^	
Baseline WC = 108.0 cm											−1.67 (−3.33 to 0.00)^a^	
Baseline WC = 118.1 cm											−3.90 (−6.73 to −1.07)^a^	
Weight *(kg)*	56	113.44 ± 32.73	108.21 ± 29.87	−5.23 ± 5.16	**<0**.**001**	54	98.00 ± 21.99	96.08 ± 21.55	−1.92 ± 3.54	**0**.**006**	−1.69 (−3.25 to −0.13)	**0**.**115**
Body mass index *(kg/m^2^*)	56	38.82 ± 9.70	37.08 ± 8.96	−1.73 ± 1.63	**<0**.**001**	53	34.33 ± 6.30	33.63 ± 5.94	−0.70 ± 1.28	**0**.**006**	−0.53 (−1.07 to 0.02)	0.115
Abdomen circumference *(cm)*	57	125.75 ± 20.51	120.38 ± 19.40	−5.36 ± 4.66	**<0**.**001**	54	116.06 ± 13.58	114.02 ± 13.57	−2.04 ± 3.75	**<0**.**001**	−2.59 (−4.15 to −1.02)	**0**.**012**
Hip circumference *(cm)*	57	125.78 ± 18.52	121.65 ± 17.15	−4.13 ± 5.70	**<0**.**001**	54	118.30 ± 13.19	116.42 ± 12.86	−1.88 ± 3.81	**<0**.**001**	−1.48 (−3.25 to 0.28)	0.159
Triceps skinfold thickness *(mm)*	57	28.09 ± 12.32	25.34 ± 10.59	−2.75 ± 6.80	**0**.**003**	54	21.53 ± 8.56	20.38 ± 8.84	−1.15 ± 5.39	0.126	0.73 (−1.68 to 3.13)	0.629
Biceps skinfold thickness *(mm)*	57	24.98 ± 10.79	22.58 ± 9.66	−2.40 ± 7.00	**0**.**012**	54	19.03 ± 5.86	18.41 ± 6.78	−0.62 ± 4.59	0.331	0.05 (−2.10 to 2.20)	0.960
Subscapular skinfold thickness *(mm)*	57	37.84 ± 11.12	34.71 ± 10.12	−3.13 ± 6.21	**<0**.**001**	54	31.62 ± 7.01	30.63 ± 8.12	−0.99 ± 3.93	0.079	−1.86 (−3.73 to 0.02)	0.115
Crista iliaca skinfold thickness (mm)	57	32.25 ± 10.76	28.52 ± 8.54	−3.73 ± 5.12	**<0**.**001**	54	27.17 ± 7.56	26.21 ± 8.21	−0.96 ± 5.56	0.218	−1.48 (−3.40 to 0.45)	0.175
Body composition parameters												
Body fat mass *(kg)*	55	51.02 ± 21.03	47.27 ± 19.92	−3.75 ± 3.71	**<0**.**001**	53	40.00 ± 13.25	37.96 ± 13.23	−2.04 ± 2.54	**<0**.**001**	−0.85 (−2.12 to 0.41)	0.273
Percent body fat *(%)*	56	43.52 ± 8.09	42.07 ± 8.66	−1.45 ± 1.59	**<0**.**001**	54	40.15 ± 7.44	38.62 ± 8.02	−1.53 ± 2.23	**<0**.**001**	0.01 (−0.81 to 0.83)	0.989
Fat-free mass *(kg)*	55	63.76 ± 16.93	62.55 ± 15.99	−1.21 ± 2.20	**<0**.**001**	53	57.56 ± 12.46	58.35 ± 12.88	0.80 ± 4.10	0.312	−1.35 (−2.54 to −0.17)	**0**.**052**
Fat-free mass index *(kg/m^2^)*	55	21.48 ± 3.67	21.08 ± 3.42	−0.41 ± 0.68	**<0**.**001**	53	20.30 ± 2.76	20.36 ± 2.66	0.07 ± 0.65	0.461	−0.29 (−0.53 to −0.04)	**0**.**052**
Skeletal muscle mass *(kg)*	56	35.42 ± 9.75	34.72 ± 9.34	−0.70 ± 1.14	**<0**.**001**	54	32.46 ± 7.51	32.64 ± 7.49	0.19 ± 1.12	0.227	−0.65 (−1.06 to −0.23)	**0**.**014**
Visceral fat area (cm^2^)	55	207.17 ± 64.23	197.90 ± 62.34	−9.26 ± 16.20	**<0**.**001**	53	194.65 ± 53.34	184.66 ± 55.15	−9.99 ± 11.07	**<0**.**001**	0.12 (−5.36 to 5.61)	0.989

Pre, baseline value; Post, day−90-standardized follow-up value; *Δ*, descriptive pre–post change. Pre–Post *p*-values, refer to within-group pre–post comparisons; AACD, adjusted active–control difference; 95% CI, 95% confidence interval; Adjusted *p*-value, *p*-value from the baseline-, age, and sex-adjusted ANCOVA model with HC3-robust inference. For secondary outcomes, the values in the final column are Benjamini–Hochberg-adjusted *p*-values calculated within the corresponding outcome family. For waist circumference, the value refers to the group-by-baseline WC interaction *p*-value and was not subject to BH adjustment**.**

a For waist circumference, the *p*-value refers to the group-by-baseline WC interaction; starred AACDs are conditional adjusted active–control differences estimated at the 25th percentile, median, and 75th percentile of baseline WC.

Bold *p*-values indicate statistically significant differences between the active and control groups at baseline (*p* < 0.05).

The study examined secondary outcomes, particularly anthropometric parameters, several of which showed significant within-group changes. Body weight and BMI decreased significantly in both groups, (−5.23 ± 5.16 kg vs. −1.92 ± 3.54 kg; *p* < 0.001; and −1.73 ± 1.63 kg/m^2^ vs. −0.70 ± 1.28 kg/m^2^; *p* = 0.006, respectively), with a significant adjusted between-group difference for body weight (*p* = 0.035), which did not remain statistically significant after Benjamini–Hochberg adjustment (*p* = 0.115). The between-group difference for BMI was not significant. Abdominal circumference decreased significantly in both the active and control groups (−5.36 ± 4.66 cm; *p* < 0.001 and −2.04 ± 3.75 cm; *p* < 0.001, respectively). The adjusted active–control difference was −2.59 cm (95% CI −4.15 to −1.02; BH-adjusted *p* = 0.012). Hip circumference decreased significantly in both the active and control groups (−4.13 ± 5.70 cm; *p* < 0.001, and −1.88 ± 3.81 cm; *p* < 0.001, respectively), but the adjusted between-group difference was not significant.

The triceps skinfold thickness decreased significantly only in the active group (−2.75 ± 6.80 mm, *p* = 0.003) compared with a descriptive change of −1.15 ± 5.39 mm (*p* = 0.126). Similarly, biceps skinfold thickness decreased significantly only in the active group (−2.40 ± 7.00 mm; *p* = 0.012), while the reduction in the control group was not significant (−0.62 ± 4.59 mm; *p* = 0.331). Subscapular skinfold also decreased significantly only in the active group (−3.13 ± 6.21 mm; *p* < 0.001), with no significant change in the control group (−0.99 ± 3.93 mm; *p* = 0.079). Similarly, crista iliaca skinfold decreased significantly in the active group (−3.73 ± 5.12 mm; *p* < 0.001), while no significant reduction was observed in the control group (−0.96 ± 5.56 mm; *p* = 0.218). However, none of the adjusted between-group differences in skinfold thickness were significant (Table 2).

When analyzing the body composition parameters, both BFM and PBF decreased in the active group (−3.75 ± 3.71 kg; *p* < 0.001 and −1.45 ± 1.59%; *p* < 0.001, respectively) and in the control group (−2.04 ± 2.54 kg; *p* < 0.001 and −1.53 ± 2.23%; *p* < 0.001, respectively). Neither adjusted between-group differences was significant. FFM, FFMI, and SMM decreased significantly in the active group (−1.21 ± 2.20 kg, *p* < 0.001; −0.41 ± 0.68 kg/m^2^, *p* < 0.001; and −0.70 ± 1.14 kg, *p* < 0.001, respectively), while the changes were not significant in the control group (0.80 ± 4.10 kg; *p* = 0.312; 0.07 ± 0.65 kg/m^2^; *p* = 0.461, and 0.19 ± 1.12 kg; *p* = 0.227). The adjusted between-group differences were nominally significant for FFM, FFMI, and SMM. After Benjamini–Hochberg adjustment, only the difference in SMM remained statistically significant (*p* = 0.014), whereas the differences in FFM and FFMI did not (*p* = 0.052 for both). VFA decreased significantly in both the active (−9.26 ± 16.20 cm^2^; *p* < 0.001) and control groups (−9.99 ± 11.07 cm^2^; *p* < 0.001). The adjusted active–control difference was 0.12 cm^2^ (95% CI −5.36 to 5.61 cm^2^; *p* = 0.965), indicating no significant between-group difference.

Considering the functional capacity data (Table 3), time to maximal exercise capacity increased significantly in the active group (+53.87 ± 168.02 s; *p* = 0.038), whereas the change was not significant in the control group (+31.75 ± 117.46 s; *p* = 0.062). Maximal MET increased significantly in the control group (+0.56 ± 1.47; *p* = 0.010), whereas the change in the active group was not significant (+0.30 ± 2.01; *p* = 0.322). In the adjusted analyses, neither time to maximal exercise capacity nor maximal MET differed significantly between the active and control groups. Regarding laboratory parameters, the mean decrease in HbA1c levels from baseline to post-intervention in the active group was −0.41 ± 0.60% (*p* < 0.001). In the control group, the mean decrease in HbA1c was −0.12 ± 0.82%, which did not reach statistical significance (*p* = 0.325). The adjusted active–control difference was −0.40 percentage points (95% CI −0.68 to −0.13; BH-adjusted *p* = 0.024). Glucose levels decreased by −0.53 ± 1.41 mmol/L, (*p* = 0.024) in the active group and by −0.52 ± 1.61 mmol/L (*p* = 0.021) in the control group. The adjusted between-group difference was not significant. Total cholesterol did not change significantly in either the active group (+0.11 ± 0.76 mmol/L; *p* = 0.287) or the control group (−0.14 ± 0.71 mmol/L; *p* = 0.160). The adjusted active–control difference was not statistically significant (0.21 mmol/L; 95% CI −0.08 to 0.50; *p* = 0.145). HDL-cholesterol increased significantly in the active group (+0.07 ± 0.13 mmol/L; BH-adjusted *p* = 0.030), while no significant change occurred in the control group (0.00 ± 0.14 mmol/L; *p* = 0.958). The adjusted active–control difference was 0.07 mmol/L (95% CI 0.02 to 0.12; *p* = 0.010). LDL-cholesterol showed no significant changes in either the active (+0.07 ± 0.56 mmol/L; *p* = 0.374) or control group (−0.14 ± 0.62 mmol/L; *p* = 0.132), and the adjusted between-group difference was not significant. Triglycerides decreased significantly only in the active group (−0.31 ± 0.67 mmol/L; *p* = 0.005), whereas the change was not significant in the control group (−0.32 ± 1.03 mmol/L; *p* = 0.053). The adjusted between-group difference was not significant.

**Table 3:** Outcome measures II. Functional capacity and laboratory parameters for the whole study population (active and control groups).

Parameter	Active group					Control group					Adjusted between-group analysis	
	n	Pre	Post	*Δ*	p	n	Pre	Post	*Δ*	p	AACD (95% CI)	BH-adjusted *p*-value
Functional capacity												
Time to maximal exercise capacity during the stress-ECG test (s)	46	634.72 ± 190.46	688.59 ± 200.60	53.87 ± 168.02	**0** **.** **038**	51	582.43 ± 154.35	614.18 ± 194.78	31.75 ± 117.46	0.062	13.06 (−56.87 to 82.99)	0.711
Maximal exercise capacity (METs)	45	7.62 ± 2.63	7.93 ± 2.72	0.30 ± 2.01	0.322	51	6.10 ± 2.06	6.66 ± 2.35	0.56 ± 1.47	**0**.**010**	−0.20 (−0.97 to 0.56)	0.711
Laboratory parameters												
HbA1c (%)	57	6.79 ± 1.26	6.38 ± 0.93	−0.41 ± 0.60	<0.001	53	7.15 ± 1.21	7.03 ± 1.14	−0.12 ± 0.82	0.325	−0.40 (−0.68 to −0.13)	**0**.**024**
Glucose (mmol/L)	57	6.85 ± 2.10	6.33 ± 1.62	−0.53 ± 1.41	**0**.**024**	54	7.99 ± 2.60	7.48 ± 2.34	−0.52 ± 1.61	**0**.**021**	−0.43 (−1.02 to 0.15)	0.174
Total cholesterol (mmol/L)	57	4.58 ± 1.02	4.69 ± 1.02	0.11 ± 0.76	0.287	54	4.85 ± 1.18	4.71 ± 1.22	−0.14 ± 0.71	0.160	0.21 (−0.08 to 0.50)	0.174
HDL-cholesterol (mmol/L)	57	1.17 ± 0.30	1.24 ± 0.29	0.07 ± 0.13	<0.001	53	1.33 ± 0.40	1.33 ± 0.39	0.00 ± 0.14	0.958	0.07 (0.02 to 0.12)	**0**.**030**
LDL-cholesterol (mmol/L)	55	2.65 ± 0.88	2.72 ± 0.91	0.07 ± 0.56	0.374	47	2.70 ± 1.01	2.55 ± 1.10	−0.14 ± 0.62	0.132	0.18 (−0.06 to 0.43)	0.174
Triglyceride (mmol/L)	57	2.01 ± 1.34	1.70 ± 1.31	−0.31 ± 0.67	**0**.**005**	54	2.16 ± 1.58	1.83 ± 0.92	−0.32 ± 1.03	0.053	−0.01 (−0.40 to 0.39)	0.976

Pre, baseline value; Post, day-90-standardized follow-up value; *Δ*, descriptive pre–post change; Pre–Post *p*-values, refer to within-group pre–post comparisons; AACD, adjusted active–control difference; 95% CI, 95% confidence interval; Adjusted *p*-value, *p*-value from the baseline-, age, and sex-adjusted ANCOVA model with HC3-robust inference. For secondary outcomes, the values in the final column are Benjamini–Hochberg-adjusted *p*-values calculated within the corresponding outcome family.

Bold *p*-values indicate statistically significant differences between the active and control groups at baseline (*p* < 0.05).

### Subgroup analysis of the active arm

Figure 2A illustrates changes in body weight between the initial visit and the interpolated 90-day time point. For subsequent analyses, the population of the active group was subdivided according to the relative change in body weight, following the predefined categories described earlier. Figure 2B shows changes for groups generated based on the relative body weight change: TQ1 ≥ 7%, marked by gray points; TQ2 5%–7%, marked by orange points; TQ3 2%–5%, marked by green points; and TQ4 < 2%, marked by blue points.

**Figure 2:**
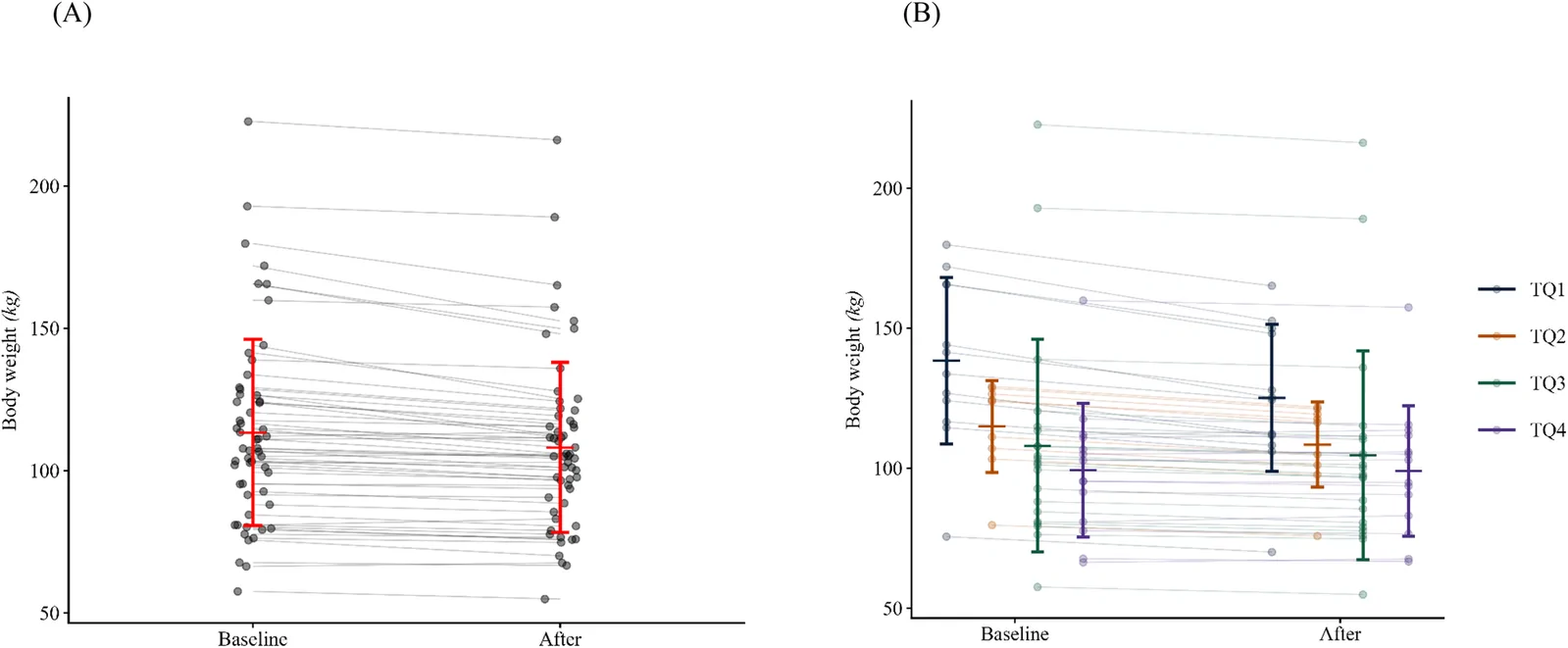
Changes in body weight in the active group between baseline and day-90-standardized data. **(A)** Whole population; **(B)** patients in TQ1, TQ2, TQ3, and TQ4 separately.

Table 4 shows the changes in body composition, functional, and laboratory parameters in the active arm across the four predefined subgroups. As expected, with increasing weight-loss effectiveness significant, linear trends were observed for WC (*p* < 0.001), AC (*p* < 0.001), and HC (*p* = 0.009) with the most pronounced changes in TQ1 group. Similarly, significant linear trends were observed for BFM (*p* < 0.001), PBF (*p* < 0.001), FFM (*p* < 0.001), FFMI (*p* < 0.001), SMM (*p* < 0.001), and VFA (*p* = 0.030) changed significantly. A significant linear trend was also observed for biceps skinfold thickness (*p* = 0.027), whereas no significant trends were observed for triceps, subscapular or crista iliaca skinfold thickness, exercise time or laboratory parameters, such as glucose, HbA1c, HDL-cholesterol, changes in triglycerides.

**Table 4:** Changes of anthropometric, body composition, functional as well as laboratory parameters for the four predefined subgroups, corrected to 90 days.

	TQ1 (*n* = 11)	TQ2 (*n* = 9)	TQ3 (*n* = 20)	TQ4 (*n* = 16)	Linear trend *β* (95%CI)	*P*-values						
						Linear trend	TQ1-TQ2	TQ1-TQ3	TQ1-TQ4	TQ2-TQ3	TQ2-TQ4	TQ3-TQ4
Anthropometric parameters												
Absolute weight *(kg)*	−13.54 ± 4.50	−7.04 ± 1.15	−3.53 ± 1.13	−0.63 ± 1.62	4.20 (3.31 to 5.09)	**<0**.**001**	**<0**.**001**	**<0**.**001**	**<0**.**001**	**<0**.**001**	**<0**.**001**	**<0**.**001**
Relative body weight *(%)*	−9.59 ± 1.93	−5.80 ± 0.55	−3.49 ± 0.97	−0.47 ± 1.51	2.96 (2.53 to 3.40)	**<0**.**001**	**<0**.**001**	**<0**.**001**	**<0**.**001**	**<0**.**001**	**<0**.**001**	**<0**.**001**
Body mass index *(kg/m^2^)*	−4.25 ± 1.41	−2.40 ± 0.65	−1.25 ± 0.38	−0.22 ± 0.57	1.32 (1.04 to 1.60)	**<0**.**001**	**<0**.**001**	**<0**.**001**	**<0**.**001**	**<0**.**001**	**<0**.**001**	**<0**.**001**
Waist circumference *(cm)*	−13.90 ± 9.49	−6.65 ± 3.95	−2.63 ± 3.33	−1.16 ± 3.40	4.17 (2.31 to 6.03)	**<0**.**001**	0.066	**0**.**003**	**<0**.**001**	**0**.**044**	**0**.**007**	0.214
Abdomen circumference *(cm)*	−10.73 ± 3.90	−7.31 ± 3.55	−4.37 ± 4.27	−1.97 ± 1.90	2.91 (2.09 to 3.73)	**<0**.**001**	0.115	**<0**.**001**	**<0**.**001**	0.115	**<0**.**001**	0.100
Hip circumference *(cm)*	−8.95 ± 6.46	−3.16 ± 4.14	−3.05 ± 5.53	−2.34 ± 4.55	1.96 (0.51 to 3.42)	**0**.**009**	0.101	0.088	**0**.**042**	>0.999	>0.999	>0.999
Triceps skinfold thickness *(mm)*	−4.88 ± 5.80	−2.52 ± 4.57	0.13 ± 6.65	−3.89 ± 6.74	0.48 (−1.13 to 2.09)	0.551	>0.999	0.243	>0.999	0.948	>0.999	0.441
Biceps skinfold thickness *(mm)*	−5.68 ± 4.88	−4.93 ± 8.16	0.14 ± 8.29	−2.08 ± 4.82	1.53 (0.18 to 2.88)	**0**.**027**	>0.999	0.128	0.374	0.593	>0.999	>0.999
Subscapular skinfold thickness *(mm)*	−5.13 ± 6.30	−5.29 ± 6.20	−0.46 ± 6.22	−4.02 ± 5.62	0.75 (−0.81 to 2.31)	0.341	>0.999	0.374	>0.999	0.374	>0.999	0.374
Crista iliaca skinfold thickness *(mm)*	−6.79 ± 8.24	−3.66 ± 2.82	−2.18 ± 3.77	−3.74 ± 4.49	1.02 (−0.68 to 2.71)	0.234	>0.999	0.591	>0.999	>0.999	>0.999	>0.999
Body composition parameters												
Body fat mass *(kg)*	−9.05 ± 3.61	−5.17 ± 1.42	−2.70 ± 1.83	−0.69 ± 1.60	2.75 (2.02 to 3.48)	**<0**.**001**	**0**.**003**	**<0**.**001**	**<0**.**001**	**0**.**002**	**<0**.**001**	**0**.**003**
Percent body fat *(%)*	−2.77 ± 1.78	−1.75 ± 1.07	−1.41 ± 1.46	−0.43 ± 1.20	0.74 (0.36 to 1.12)	**<0**.**001**	0.280	0.141	**0**.**004**	0.502	**0**.**044**	0.141
Fat-free mass *(kg)*	−4.02 ± 2.62	−1.95 ± 0.96	−0.54 ± 1.13	0.25 ± 1.38	1.41 (0.87 to 1.95)	**<0**.**001**	0.053	**<0**.**001**	**<0**.**001**	**0**.**008**	**<0**.**001**	**0**.**079**
Fat-free mass index *(kg/m^2^)*	−1.21 ± 0.78	−0.71 ± 0.37	−0.19 ± 0.40	0.03 ± 0.47	0.42 (0.26 to 0.59)	**<0**.**001**	0.173	**0**.**001**	**<0**.**001**	**0**.**010**	**0**.**001**	**0**.**173**
Skeletal muscle mass *(kg)*	−1.89 ± 1.59	−1.09 ± 0.61	−0.36 ± 0.66	−0.09 ± 0.78	0.61 (0.29 to 0.93)	**<0**.**001**	0.302	**0**.**020**	**0**.**008**	**0**.**022**	**0**.**008**	0.302
Visceral fat area (cm^2^)	−20.41 ± 24.50	−9.44 ± 15.80	−8.23 ± 12.82	−2.81 ± 9.11	5.43 (0.56 to 10.30)	**0**.**030**	0.802	0.738	0.206	0.855	0.802	0.738
Functional capacity												
Exercise time to maximal exercise capacity during the stress-ECG test (s)	91.68 ± 80.43	38.22 ± 66.06	66.08 ± 201.58	−3.71 ± 180.60	−27.70 (−63.46 to 8.05)	0.125	>0.999	>0.999	0.605	>0.999	>0.999	>0.999
Laboratory parameters												
Glucose *(mmol/L)*	−0.03 ± 0.99	−0.85 ± 2.39	−0.67 ± 1.38	−0.58 ± 0.97	−0.14 (−0.44 to 0.15)	0.341	>0.999	0.933	0.933	>0.999	>0.999	>0.999
HbA1c *(%)*	−0.31 ± 0.44	−0.69 ± 0.82	−0.39 ± 0.63	−0.28 ± 0.48	0.04 (−0.09 to 0.17)	0.527	>0.999	>0.999	>0.999	>0.999	>0.999	>0.999
HDL-cholesterol *(mmol/L)*	0.05 ± 0.12	0.06 ± 0.09	0.06 ± 0.12	0.11 ± 0.18	0.02 (−0.02 to 0.06)	0.303	>0.999	>0.999	>0.999	>0.999	>0.999	>0.999
Triglyceride *(mmol/L)*	−0.40 ± 0.50	−0.39 ± 0.69	−0.08 ± 0.38	−0.50 ± 0.98	−0.01 (−0.19 to 0.18)	0.952	>0.999	0.531	>0.999	0.962	>0.999	0.634

Bold *p*-values indicate statistically significant differences between the active and control groups at baseline (*p* < 0.05).

Analysis of adherence-related parameters in the active group, subdivided by relative weight change (TQ1–TQ4), is summarized in Table 5. The ratio of days with food records (Food_count_per_duration) was slightly higher in TQ1 (0.80 ± 0.09) compared with TQ4 (0.69 ± 0.25), but the linear trend was not statistically significant (*p* = 0.129). No significant linear trend was observed for the ratio of days with Polar-recorded physical activity (Polar_count_per_duration; *p* = 0.394). In contrast, the ratio of days with weight records (Weight_count_per_duration) showed a significant linear trend across the TQ1–TQ4 subgroups, with TQ1 showing higher adherence than TQ3 (*p* < 0.001) and TQ4 (*p* < 0.001). Average daily calorie intake relative to BMR (Food_kcal_mean_per_bmr) also showed a significant linear trend (*p* < 0.001), with TQ1 recording lower relative intake than TQ3 (*p* < 0.002) and TQ4 (*p* = 0.011). No significant linear trend was observed for average daily training-related energy expenditure relative to BMR (Polar_kcal_mean_per_bmr; *p* = 0.481). These findings indicate that more frequent body weight recording and lower energy intake relative to BMR were associated with greater body-weight loss, whereas no significant linear trends were observed for the other adherence parameters.

**Table 5:** Monitoring adherence and changes in monitored parameters across subgroups.

Adherence parameter	TQ1	TQ2	TQ3	TQ4	Linear trend *β* (95%CI)	*P*-values						
	*(n* *=* *11)*	*(n* *=* *9)*	*(n* *=* *20)*	*(n* *=* *16)*		Linear trend	TQ1 vs. TQ2	TQ1 vs. TQ3	TQ1 vs. TQ4	TQ2 vs. TQ3	TQ2 vs. TQ4	TQ3 vs. TQ4
Food_count_per_duration	0.80 ± 0.09	0.76 ± 0.20	0.74 ± 0.27	0.69 ± 0.25	−0.04 (−0.08 to 0.01)	0.129	>0.999	>0.999	0.750	>0.999	>0.999	>0.999
Polar_count_per_duration	0.47 ± 0.25	0.56 ± 0.25	0.49 ± 0.25	0.41 ± 0.22	−0.03 (−0.09 to 0.03)	0.394	>0.999	>0.999	>0.999	>0.999	0.956	>0.999
Weight_count_per_duration	0.93 ± 0.08	0.79 ± 0.24	0.65 ± 0.27	0.62 ± 0.23	−0.11 (−0.15 to −0.06)	**<0**.**001**	0.427	<0.001	<0.001	0.427	0.427	0.728
Food_kcal_mean_per_bmr	0.60 ± 0.14	0.70 ± 0.13	0.84 ± 0.17	0.86 ± 0.26	0.09 (0.04 to 0.14)	**<0**.**001**	0.246	<0.001	0.011	0.097	0.164	0.798
Polar_kcal_mean_per_bmr	0.23 ± 0.13	0.30 ± 0.32	0.26 ± 0.22	0.20 ± 0.13	−0.01 (−0.05 to 0.03)	0.481	>0.999	>0.999	>0.999	>0.999	>0.999	>0.999

Bold *p*-values indicate statistically significant differences between the active and control groups at baseline (*p* < 0.05).

## Discussion

### Primary outcome: waist circumference

Our prospective controlled study examined the impact of telemedicine-based dietary and physical training interventions on patients with MetS. Despite the challenges posed by the COVID-19 pandemic, which necessitated adjustments in study design and recruitment methods, our findings suggest that a telemedicine-supported lifestyle intervention may improve selected anthropometric outcomes in patients with MetS. The −5.0 cm mean reduction in WC (primary outcome) in the active group is clinically relevant. While both groups demonstrated some degree of improvement due to the heightened awareness engendered by study participation, the active group exhibited greater improvement, although the non-randomized design and baseline differences necessitate cautious interpretation. The result of the present study is greater than the WC reduction of 1.1–3.4 centimeters that was achieved in our previous telerehabilitation study, which incorporated identical heart rate-based exercise telemonitoring for a duration of 12 weeks, but did not encompass nutrition coaching (16). It also exceeds the result of Ross et al. (32), who performed a 24-week intervention with three structured exercise sessions per week regimens yielding WC reductions in the range of −3.9 to −4.6 cm. The larger within-group reduction may reflect differences in intervention intensity, study population, or study design, but direct comparisons between studies should be interpreted cautiously.

### Secondary outcome: body weight

Weight loss in the intervention group reached an average of −5.2 kg over 12 weeks, surpassing the −1.9 kg observed in the sequential control group. The magnitude of this change is consistent with the classical LEARN behavioral program, which produced approximately 6 kg weight loss in a similar 12-session format (33). By contrast, lower-intensity digital interventions — such as informational or app-based counseling without consistent logging or personalized feedback — tend to produce smaller changes. A customized digital health care service (CDHCS) delivered over 3 months generated small, non-significant reductions in weight and WC (34), and the eMOTIVA mHealth program for post-acute coronary syndrome patients also only documented improved diet quality and physical activity without reporting meaningful short-term weight change (35). When viewed across this spectrum, the magnitude of weight loss observed in our intervention group was within the upper range reported for behavioral and telehealth-supported programs of similar duration, although indirect comparisons between studies should be interpreted cautiously. Under this model, participants achieved −5.2 kg weight loss in 12 weeks, suggesting that this multimodal approach may support clinically meaningful short-term weight reduction.

A key challenge limiting most lifestyle-intervention programs is that they cannot deliver a truly full-spectrum intervention, largely because commercially available diet-logging applications operate as standalone market products that healthcare providers are typically unable to integrate into their clinical systems. A distinctive strength of the present intervention was the seamless integration of a diet-logging application within the telemedical workflow.

### Secondary outcome: body composition

Changes in body weight in the present study were accompanied by favorable changes in body composition. Fat mass decreased by approximately −3.7 kg, though a concurrent and significant decrease in fat-free mass (FFM, −1.2 kg), and skeletal muscle mass (SMM, −0.7 kg) was also documented. The type and intensity of digital interventions play a crucial role in these outcomes. While low-intensity apps or educational websites often produce parallel shifts in fat and lean mass without selective fat reduction (34, 36), the multimodal telemedical approach used in this study — incorporating granular feedback loops and expert oversight — facilitated clinically meaningful shifts in composition.

The observed FFM reduction represents approximately 23% of total weight loss, which is consistent with the roughly 25% ratio typically reported in classic hypocaloric diet programs (37, 38). While supervised exercise can attenuate lean mass loss (39), our findings suggest that an aerobic-focused protocol may not be sufficient for full tissue preservation (40).

Although higher activity levels (250–300 min/week) and resistance exercises are linked to better maintenance of FFM (41, 42), our program specifically targeted the standard 150-minute weekly cardio goal through heart rate-based monitoring. This technological focus on aerobic capacity likely contributed to the observed muscle loss, highlighting the need for future telerehabilitation systems to move beyond cardio-centric monitoring and better integrate structured, telemonitored resistance training.

In the contemporary era, when pharmacological approaches such as GLP-1 receptor agonists are increasingly used for body weight reduction, it is informative to place our findings in the context of previously published data. Based on recent publications, this group of drugs is capable of inducing a considerable weight loss of 8–15 kg over 6–12 months, with an estimated 60%–75% of this reduction arising from fat mass loss (approximately 6–11 kg), alongside an absolute decrease in fat-free mass of around 1–3 kg (43, 44). While our 12-week lifestyle intervention resulted in a smaller absolute magnitude of weight loss, the observed ratio of fat-to-lean mass reduction remains a clinically meaningful component of MetS management. These results suggest that intensive telemedical support can serve as a valuable non-pharmacological strategy, although the non-randomized nature of this study and the observed muscle loss necessitate cautious comparison with established pharmacological outcomes.

### Outcome and adherence

In our study, the subgroup analyses identified factors associated with more favourable outcomes. The number of days with body weight logging (self-weighing) was significantly associated with relative weight loss, however, the observational nature of this analysis does not establish a causal relationship. We could not document an association of similar strength for food or exercise logging. A correlation between bodyweight measurement frequency and the change of body-weight was earlier also documented in an analysis of a large vendor-specific non-interventional database consisting of more than 10,000 users (45). They found that the percentage of self-weighing days correlated inversely with weight change, r = −0.111 (*p* < 0.001). During the breaks with ≥30 days without weight measurements, weight increased.

Liu et al. (46), in the MOVE UP trial, documented a correlation also between lifestyle log submission adherence and weight-loss, but they investigated an older population then the population in our study. Similarly, Nagpal et al. (47) documented that higher adherence scores — derived by grading participants’ weekly achievement of predefined nutrition and exercise goals within the NELIP program — were significantly associated with more favorable gestational weight outcomes.

Burgess et al. underscored in their review (48) the heterogeneity and limitations of adherence assessment across lifestyle trials: many studies assessed only session attendance rate, but most did not evaluate whether participants adhered to the prescribed caloric targets or exercise intensity. Dietary adherence was quantified in only two of their reviewed studies, and self-monitoring was typically assessed only by the number of logs completed rather than the accuracy or fidelity of logged behaviours. This aligns with our findings, where detailed food-diary content — but not merely logging frequency — correlated with body-weight change, illustrating that attendance alone cannot capture the true behavioural engagement within an intervention. Interestingly, we were unable to detect a comparable association between objectively measured exercise intensity and short-term body-weight change. This is physiologically plausible, as exercise tends to exert its strongest effects on the preservation and accrual of lean mass, on cardiometabolic fitness, and on long-term risk reduction, whereas its isolated contribution to early weight loss is typically more modest.

In our study, patients with metabolic syndrome in the active intervention group demonstrated improvements in laboratory parameters such as HbA1c and HDL-cholesterol, indicating enhanced metabolic control. Although telemedicine-based lifestyle interventions consistently demonstrate beneficial effects on anthropometric outcomes, including body weight and waist circumference, their effects on metabolic laboratory markers remain less uniform. Several systematic reviews have reported robust improvements in anthropometric measures but only modest or inconsistent changes in glycemic control and lipid profiles (49, 50). Chen et al. (51) reported that eHealth interventions consistently improved anthropometric outcomes but showed no significant or consistent effects on most biochemical parameters. In contrast, Sequi-Domínguez et al. (52) observed significant improvements not only in weight and waist circumference but also in certain biochemical markers such as fasting plasma glucose and HDL-cholesterol.

### Limitations

The main limitation of this study is that it was performed during the COVID-19 pandemic, when telemedicine was highly relevant; however, face-to-face visits for control participants would have posed an unjustified risk. Since the course of the pandemic was unpredictable at this time, the investigators of this study decided to break the study architecture in this force majeure situation, undertake evaluation only of the telemedical study arm and postpone the involvement of control patients until the subsiding of the pandemic, generating a sequential control arm. Recognizing the baseline differences between the study arms due to the non-randomized recruitment, we addressed these discrepancies through statistical adjustment. We intentionally utilized a parsimonious correction model (ANCOVA adjusted for baseline values, age, and sex) to strike a balance between enhancing comparability and avoiding the introduction of statistical artifacts or the inadvertent masking of real effects. This approach allows for a more robust evaluation of the intervention within the constraints of the study design. Given the promising initial results, we believe these findings offer important pilot-scale evidence that justifies further investigation in a routine clinical setting. Unfortunately, we have significantly less evidence regarding the effectiveness of lifestyle interventions than we do for new drugs. As lifestyle interventions are generally not patentable, funding for large-scale randomized trials is often limited. We must greatly value the evidence that we have been able to produce with community support. Consequently, utilizing real-world evidence frameworks is a pragmatic way to assess the effectiveness of such promising methods in routine practice. Based on the above results, the University of Szeged has launched a pilot project to evaluate this method further. We hope that this initiative will enable us to answer questions in the near future based on real-world data that could not be addressed due to the scope of the present study.

The intervention period applied in this study (90 days) was relatively short; while improvements were measurable, the long-term sustainability of these benefits remains uncertain. Furthermore, the COVID-19 pandemic introduced significant variability in follow-up assessments, which were performed between 83 and 178 days after enrollment. To account for this heterogeneity, we standardized clinical values to a 90-day period using linear calculation. We acknowledge that this method assumes a constant average rate of change, which may not fully capture the distinct biological trajectories, such as different rates of weight loss or metabolic shifts in early versus late phases. However, there is methodological precedent for using time-proportional standardization in longitudinal weight and metabolic research, such as the approaches utilized by Forouhi et al. and Paige et al. (53, 54).

Although telemonitoring (weight scale, app, workout tracker) reduces reliance on in-person visits, self-reported dietary intake may still be biased by under- or over-reporting.

Finally, our sample may not generalize broadly: participants with sufficient digital literacy and motivation to enroll in a telemedical program may differ systematically from the general MetS population.

## Conclusion

The findings of this study support the feasibility and potential clinical utility of telemedicine-supported lifestyle intervention for patients with MetS. The observed short-term improvements in selected anthropometric and metabolic outcomes warrant further evaluation in larger, randomized studies with concurrent control groups and longer follow-up. Such studies are also needed to assess generalizability, scalability, sustainability, and cost-effectiveness.

Our data suggest that a telemedicine-supported lifestyle program may contribute to short-term improvements in selected metabolic risk factors in patients with MetS. Specifically, regular self-weighing/self-monitoring emerged as a practical component that is associated with greater weight loss. Since digital platforms facilitate broader access, such models are particularly well-suited for resource-limited settings or for patients with limited time availability and mobility.

## Data Availability

The raw data supporting the conclusions of this article will be made available by the authors, without undue reservation.
